# NF-kappa B activation correlates with disease phenotype in Crohn’s disease

**DOI:** 10.1371/journal.pone.0182071

**Published:** 2017-07-28

**Authors:** Yoo Min Han, Jaemoon Koh, Ji Won Kim, Changhyun Lee, Seong-Joon Koh, ByeongGwan Kim, Kook Lae Lee, Jong Pil Im, Joo Sung Kim

**Affiliations:** 1 Department of Internal Medicine and Healthcare Research Institute, Seoul National University Hospital Healthcare System Gangnam Center, Seoul, Korea; 2 Department of Pathology, Seoul National University College of Medicine, Seoul, Korea; 3 Department of Internal Medicine, Seoul National University College of Medicine, Seoul, Korea; 4 Department of Internal Medicine, Seoul National University Boramae Hospital, Seoul, Korea; Universitatsklinikum Aachen, GERMANY

## Abstract

**Background/Aims:**

Unregulated activation of nuclear factor-κB (NF-κB) plays a critical role in the pathogenesis of Crohn’s disease. In this study, we investigated the clinical characteristics and disease outcome of Crohn’s disease patients with varying levels of the NF-κB activation.

**Methods:**

Crohn’s disease patients who underwent surgical bowel resection were divided into two groups, based on the activation status of NF-κB. NF-κB activation was assessed by the immunoreactivity of nuclear NF-κB during immunohistochemical staining of bowel resection specimens. We compared the demographic, clinical and histologic characteristics between groups. Furthermore, the occurrence of reoperation, readmission, and medication change due to disease flare-up were investigated according to NF-κB activation status.

**Results:**

Among 83 Crohn’s disease patients, 47 (56%) showed high NF-κB activity and 36 (44%) showed low NF-κB activity. Patients with high NF-κB activity had higher frequency of ileocolonic involvement (P = 0.028) and lower frequency of perianal involvement (P = 0.042) relative to those with low NF-κB activity. Total histologic scores were significantly higher in patients with high NF-κB activity than those with low NF-κB activity (P = 0.044). There was no significant difference in the frequency of reoperation, readmission, and medication change in relation to NF-κB activation status.

**Conclusions:**

Crohn’s disease patients with high NF-κB activation showed specific clinical manifestations of higher frequency of ileocolonic involvement and lower frequency of perianal involvement relative to those with low NF-κB activation. High NF-κB activity was associated with higher histologic scores. However, the NF-κB activity did not affect the outcome and disease course after surgery.

## Introduction

Crohn’s disease (CD) is a chronic inflammatory disease of unknown etiology that involves the entire digestive tract (mouth to anus). The mechanism of CD pathogenesis is unclear, although factors, such as genetic predisposition, immunologic imbalance, and host-microbial interaction, are known to be involved [1, 2]. CD is most prevalent in North America and Europe, though the incidence in Asian countries is rapidly increasing [3, 4].

Abnormal responses to intestinal microorganisms and dysregulation of nuclear factor-κB (NF-κB) signaling pathways are associated with disease initiation and progression [5, 6]. Abnormal activation of NF-κB leads to excessive production of pro-inflammatory cytokines that cause chronic inflammation in the bowel. In a previous report, patients with inflammatory bowel disease (IBD) showed significantly higher expression rates of NF-κB when compared with non-IBD patients [7, 8]. Furthermore, a significant increase in the number of NF-κB positive cells was noted on performing immunostaining in the inflamed area compared with the non-inflamed areas in the same CD patients [8]. As the NF-κB pathway plays a key role in the pathology of IBD, current therapies aim to block this pathway. A number of previous experiments have shown that NF-κB activation decreases after treatment with anti-IBD medications such as 5-aminosalycilic acid and corticosteroids [9–11]. These studies suggest that NF-κB activation status might reflect the inflammatory burden in CD and it could be closely correlated with disease activity.

Patients with IBD undergo heterogeneous disease courses, some have mild and relapse free disease, while others have chronic and recurrent disease [12]. Several prognostic factors have been evaluated to predict disease course. In one study, the need for steroid administration in response to the first flare-up of the disease and perianal lesions at the time of diagnosis were associated with poor prognosis [13]. An age below 40 years and disease location have also been identified as risk factors for poor prognosis in previous studies [14, 15]. However, these studies mainly dealt with demographic and clinical characteristics to predict disease prognosis. To the best of our knowledge, there have been few studies which identified pathologic and molecular markers for CD prognosis.

Considering that unregulated NF-κB pathway activation plays a critical role in disease pathogenesis, we hypothesized that NF-κB activation levels influence clinical manifestation and disease course in CD patients. The aim of this study was to evaluate the association between NF-κB activation and the CD phenotype. Furthermore, we evaluated whether NF-κB activation is associated with disease outcomes of CD over time after surgical treatment.

## Materials and methods

The study was approved by the International Review Board (IRB) of Seoul National University Hospital (IRB no. H-1208-054-421) and was conducted in accordance with the Declaration of Helsinki. The IRB of Seoul National University Hospital waived the need for written consent for their medical records to be used. The data was accessed anonymously.

### Patients

CD patients, who were diagnosed and who underwent follow-up evaluation between 2005 and 2012 at Seoul National University Hospital and Seoul National University Boramae Hospital, were screened for inclusion in the present study. CD was diagnosed according to clinical, endoscopic, radiological, and histological criteria [16]. Patients who underwent surgical bowel resection related to CD, and whose pathological specimens were available, were included in this study.

Age, sex, age at diagnosis, past medical history, smoking history, and follow up duration were reviewed. In addition, extra-intestinal manifestation (oral stomatitis, cutaneous manifestation, ocular manifestation, and arthritis), extent of disease, disease behavior at diagnosis, presence of perianal lesions, and anti-tuberculosis medication history were evaluated. Medical records, including the date, location, and reason for operation, were also reviewed.

### Tissue microarray construction

Formalin-fixed, paraffin-embedded (FFPE) tissue blocks from resection specimens were obtained. The microarray consisted of tissues derived from resected specimens with a confirmed hematoxylin and eosin stain and clinical diagnosis. A 2 mm diameter tissue cylinder was punched from a morphologically representative tissue area in each tissue block. Three cores per resection specimen were obtained for each individual case. Tissues in the area accompanying fistulas or perforations were excluded, because there was a chance that severe inflammatory responses secondary to the fistula or perforation, which are unrelated to CD, could be present.

### Immunohistochemistry (IHC)

**Fig 1:**
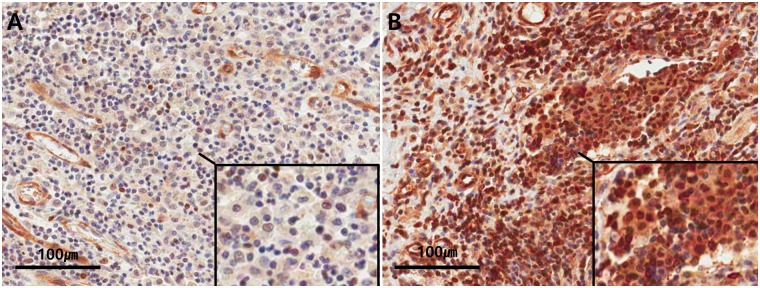
Representative IHC staining of NF-κB in CD. NF-κB positive inflammatory cells (brown) and counterstaining (blue). A. A case with a low number of NF-κB positive inflammatory cells (x400). B. A case with a high number of NF-κB positive inflammatory cells (x400).

FFPE tissue blocks were cut into 4 μm thick slices and they underwent immunostaining. Rabbit polyclonal anti-NF-κB p65 antibody (ab16502, Abcam, Cambridge, UK) staining was performed at a 1:200 dilution according to the protocols provided by the Ventana Benchmark XT automated staining system (Ventana Medical Systems, Tucson, AZ, USA) (Fig 1) and also Mayer's hematoxylin staining was performed as a counterstain.

### Interpretation of immunostained sections (scoring system)

**Fig 2:**
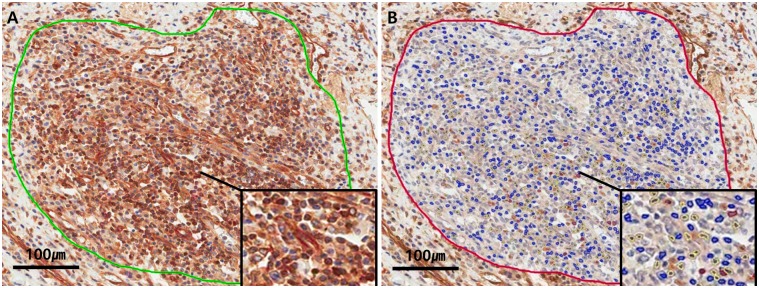
Counting NF-κB immunoreactive inflammatory cells using computed-image analysis. A. Pre-analysis. Selection of the analysis area. B. Post-analysis. Software measurement of single stain nuclear IHC positivity and classification as 1+ (yellow), 2+ (orange), 3+ (red), or negative (blue).

NF-κB slides were scanned for each tissue microarray using the Aperio ScanScope (Aperio Technologies, Vista, CA, USA). After saving each digital image, all intact areas (excluding areas of significant crush artifact, necrosis, or otherwise other poor quality) and corresponding inflammatory regions on the NF-κB slides were selected. Aperio ImageScope software (Aperio Technologies) and Aperio nuclear IHC algorithms were used for analysis of NF-κB staining (Fig 2). Because NF-κB translocates to the nucleus, where it acts as a positive regulator of target genes, only nuclear staining was counted. To specifically count lymphocytes of lamina propria, we applied the nuclear algorithm parameters, including minimum and maximum nuclear size. As a result, it was possible to count NF-κB-expressing lymphocytes selectively using nuclear IHC algorithms. The analysis algorithms were based on the spectral differentiation between brown (positive) and blue (counter) staining. The total percentage of positive cells (1+ through 3+) was recorded for each case. To avoid interpretation error, we considered only IHC scores of 2+ and 3+ to be positive, as non-specific staining could potentially causes a score of 1+. The percentage of positive cells was counted in 3 representative high power fields. The mean percentage of positive cells was divided into two categories: below 25% was classified as low NF-κB activity, and above 25% was classified as high NF-κB activity [7, 17]. Slides were scored by a single pathologist without any knowledge of patient clinical information and outcome.

### Interpretation of histologic score

Histologic assessment was performed for each slide. Seven histologic variables, which were epithelial damage (0 = normal, 1 = focal, 2 = extensive), architectural changes (0 = normal, 1 = moderate, 2 = severe), mononuclear cells in lamina propria (0 = normal, 1 = moderate increase, 2 = severe increase), polymorphonuclear cells in lamina propria (0 = normal, 1 = moderate increase, 2 = severe increase), neutrophils in epithelium (1 = surface epithelium, 2 = cryptitis, 3 = crypt abscess), erosions or ulceration (0 = no, 1 = yes), and granuloma (0 = no, 1 = yes) were evaluated. Total histologic score was calculated as sum of each score. [18, 19] Slides were scored by a single pathologist without any knowledge of patient clinical information and outcome.

### Disease severity and outcome measures

To evaluate disease prognosis, three main outcome variables were analyzed. First, data regarding reoperation due to uncontrolled CD and time to reoperation were obtained. Second, the rate of readmission due to disease flare-ups and the time to readmission from initial operation were assessed. Third, any change in CD-related therapy, including administration of a new drug class or a switch to another drug in the same class, were analyzed. These medications included systemic steroids, thiopurines (azathioprine and 6-mercaptopurine), 5-amino-salicylates (mesalazine and sulfasalazine), and anti-tumor necrosis factor alpha (TNF-α) agents (infliximab and adalimumab). We extended the duration of follow-up to the patient’s last hospital visit, and monitored both flare-up occurrences and time to flare-ups.

### Statistical analysis

The data were analyzed using SPSS version 23.0 for Windows (SPSS Inc., Chicago, IL, USA). Results were expressed as the mean ± standard deviation (SD), the median with interquartile range (IQR), or as counts with percentage. Correlation coefficients were calculated using the Student’s t-test for continuous variables and one of Pearson’s chi-squared test, Fisher’s exact test or Linear by linear association for categorical variables. Repeated measurement ANOVA method was used to check the consistency of three repetitive measurement of NF-κB activity.

The Kaplan-Meier method was used to compare the difference between long-term outcomes according to NF-κB activity. The log-rank test was used to compare the statistical differences between groups. The metrics for CD prognosis were defined as the occurrence of reoperation, readmission, and medication change. *P*-values less than 0.05 were considered statistically significant.

## Results

### Baseline characteristics

Among patients diagnosed with CD, 120 patients underwent surgical bowel resection. We excluded 13 patients from the analysis because their surgeries were not related to CD and an additional 24 patients because pathological specimens could not be obtained. Thus, 83 patients were included in this study. The age was 36.41 ± 13.54 years, and age at diagnosis was 25.24 ± 12.72 years. The male to female ratio was 59 to 24, and the median follow-up duration was 64 months (IQR 30–116 months).

### NF- κB expression and CD clinical manifestation

**Fig 3:**
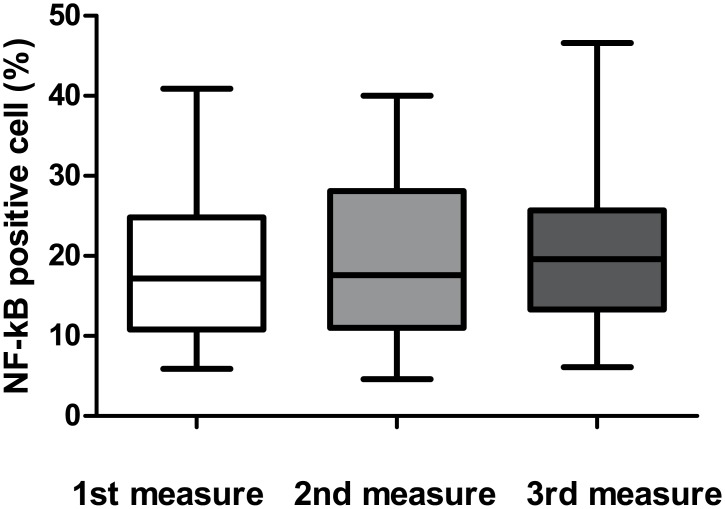
The percentage of NF-κB positive cells in three representative fields. The percentage of NF-κB positive cells at each of the three representative areas were not significantly different (18.84 ± 9.18%, 19.24 ± 9.20%, and 20.07 ± 8.92%, respectively; P = 0.371).

We assessed the NF-κB staining in three representative areas of each slide using previously reported scale [7, 17]. The percentage of positive cells were not significantly different among three areas (18.84 ± 9.18%, 19.24 ± 9.20%, and 20.07 ± 8.92%, respectively; P = 0.371, Fig 3). The mean value was 19.38% (ranged 6.93%-37.30%). So we classified score 1 (below 25%) as low NF-κB activity, and score 2 (above 25%) as high NF-κB activity.

Patients were sorted into 2 categories, with 47 patients (56.6%) in the low NF-κB activity group and 36 patients (43.4%) in the high NF-κB activity group. Demographic and clinical data are shown in Table 1. Patients with high NF-κB activity showed more frequent ileocolonic involvement (P = 0.028) and less frequent perianal involvement (P = 0.042) relative to those with low NF-κB activity. Between the two groups, there was no significant difference in age, sex, family history, extra-intestinal manifestation (oral stomatitis, cutaneous manifestation, ocular manifestation, and arthritis), disease behavior at diagnosis, smoking history, and history of anti-tuberculosis medication.

**Table 1:** Baseline characteristics of high and low NF-κB activity groups.

Characteristics	High NF-κB activity (n = 36)	Low NF-κB activity (n = 47)	*P*-value
**Median age [years (IQR)]**	36.5 (26.3–42.5)	36.0 (26.0–47.0)	0.761 †
**Median age at diagnosis [years (IQR)]**	24.5 (14.3–34.0)	22.0 (17.0–30.0)	0.787†
**Sex**			0.206 §
Male [n (%)]	23 (63.9)	36 (76.6)	
Female [n (%)]	13 (36.1)	11 (23.4)	
**Family history of CD [n (%)]**	0 (0.0)	3 (6.4)	0.254 ‡
**Extraintestinal manifestation [n (%)]**	2 (5.6)	4 (8.5)	0.693 ‡
Oral stomatitis [n (%)]	2 (5.6)	3 (6.4)	1.000 ‡
Cutaneous manifestation [n (%)]	0 (0.0)	1 (2.1)	1.000 ‡
Ocular manifestation [n (%)]	0 (0.0)	1 (2.1)	1.000 ‡
Arthritis [n (%)]	1 (2.8)	2 (4.3)	1.000 ‡
**Extent of disease**			0.028 §
L1 (terminal ileum) [n (%)]	7 (19.4)	21 (44.7)	
L2 (colon) [n (%)]	1 (2.8)	3 (6.4)	
L3 (ileocolon) [n (%)]	28 (77.8)	23 (48.9)	
L4 (upper gastrointestinal tract) [n (%)]	0 (0.0)	0 (0.0)	
**Disease behavior at diagnosis**			0.589§
B1 (NS-NP) [n (%)]	20 (55.6)	31 (66.0)	
B2 (stricturing) [n (%)]	9 (25.0)	8 (17.0)	
B3 (penetrating) [n (%)]	7 (19.4)	8 (17.0)	
**Perianal lesion [n (%)]**	9 (25.0)	22 (46.8)	0.042 §
**Smoking history [n (%)]**	4 (11.1)	5 (10.6)	1.000 †
**Anti-tuberculosis medication**			0.293 §
Yes [n (%)]	14 (38.9)	11 (23.4)	
No [n (%)]	4 (11.1)	8 (17.0)	
Unknown [n (%)]	18 (50.0)	28 (59.6)	
**Reason for primary operation**			0.767 §
Perforation [n (%)]	10 (27.8)	9 (19.1)	
Fistula [n (%)]	8 (22.2)	13 (27.7)	
Stricture [n (%)]	14 (38.9)	16 (34.0)	
Bleeding [n (%)]	0 (0.0)	1 (2.1)	
Intractable to medical therapy [n (%)]	3 (8.3)	7 (14.9)	
For diagnosis [n (%)]	1 (2.8)	1 (2.1)	

^†^Independent Student’s t-test

^‡^Fisher’s exact test

^§^Pearson’s chi square test

### NF-κB expression on immunohistochemical analysis and histologic score

**Fig 4:**
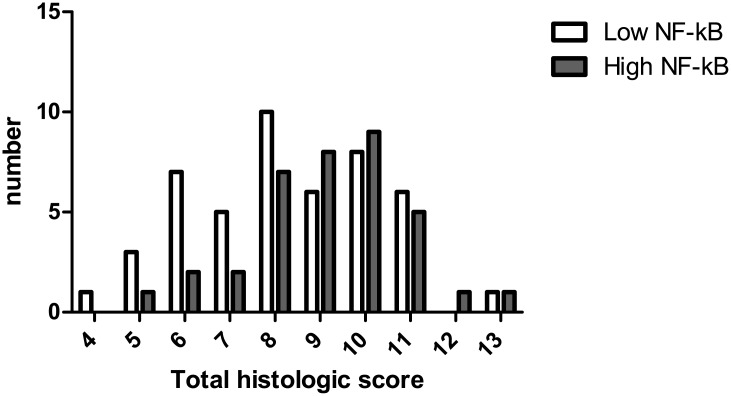
Total histologic scores in patients with high and low NF-κB activity. Total histologic scores were significantly higher in patients with high NF-κB activity than those with low NF-κB activity (P = 0.044).

Patients with high NF-κB activity showed more polymorphonuclear cells in lamina propria and neutrophils in epithelium than those with low NF-κB activity (P = 0.050 and P = 0.010, respectively). Granulomas were also more prevalent in patients with high NF-κB activity (P = 0.034). Overall histologic scores were significantly higher in patients with high NF-κB activity than those with low NF-κB activity (P = 0.044, Fig 4). Between high and low NF-κB activity groups, there was no significant difference in epithelial damage, architectural change, mononuclear cell in lamina propria, and erosions or ulcers (Table 2).

**Table 2:** Histologic scores of high and low NF-κB activity groups.

Characteristics	High NF-κB activity (n = 36)	Low NF-κB activity (n = 47)	*P*-value
**Epithelial damage**			0.165 †
1. Focal [n (%)]	26 (72.2)	(57.4)	
2. Extensive [n (%)]	10 (27.8)	20 (42.6)	
**Architectural Change**			0.536†
1. Moderate [n (%)]	19(52.8)	59.6)	
2. Severe [n (%)]	17 (47.2)	19 (40.4)	
**Mononuclear cell in lamina propria**			0.572†
1. Moderate increase [n (%)]	8 (22.2)	27.7)	
2. Severe increase [n (%)]	28 (77.8)	34 (72.3)	
**Polymorphonuclear cell in lamina propria**			0.050‡
0. Normal [n (%)]	1 (2.8)	(12.8)	
1. Moderate increase [n (%)]	25 (69.4)	72.3)	
2. Severe increase [n (%)]	10 (27.8)	7 (14.9)	
**Neutrophil in epithelium**			0.010 ‡
1. Surface epithelium [n (%)]	13 (36.1)	61.7)	
2. Cryptitis [n (%)]	17 (47.2)	34.0)	
3. Crypt abscess [n (%)]	6 (16.7)	2 (4.3)	
**Erosions or Ulcers**			0.725§
0. No [n (%)]	3 (8.3)	(12.8)	
1. Yes [n (%)]	33 (91.7)	41 (87.2)	
**Granulomas**			0.034 †
0. No [n (%)]	13 (36.1)	59.6)	
1. Yes [n (%)]	23 (63.9)	19 (40.4)	
**Total Histologic score**			0.044‡

^†^ Pearson’s chi square test

^‡^ Linear by linear association

^§^ Fisher’s exact test

### NF-κB expression on immunohistochemical analysis and CD prognosis

The Kaplan-Meier method and log rank test were used to analyze the correlation between NF-κB activation and long term prognosis. There was no significant difference in rates of reoperation, readmission, or medication change between the high and low NF-κB activity groups.

**Fig 5:**
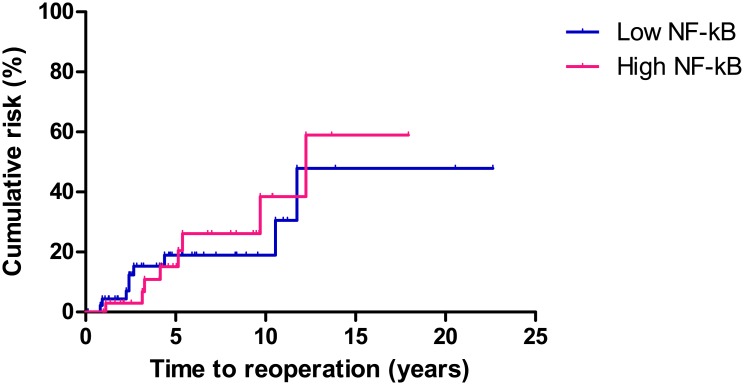
Comparison of reoperation in patients with high and low NF-κB activity. There was no significant difference in rates of reoperation between the high and low NF-κB activity groups (P = 0.840).

Reoperation occurred in 17 (20.4%) patients during the follow-up period. The median time to reoperation was 53.3 months (IQR, 27.4–101.2 months) from the primary operation. The cumulative rate of reoperation at 1, 5, and 10 years was 0.0%, 11.1%, and 19.4%, respectively, in the patients with low NF-κB activity, and 4.3%, 14.9%, and 14.9%, respectively, in the patients with high NF-κB activity (P = 0.840, Fig 5).

**Fig 6:**
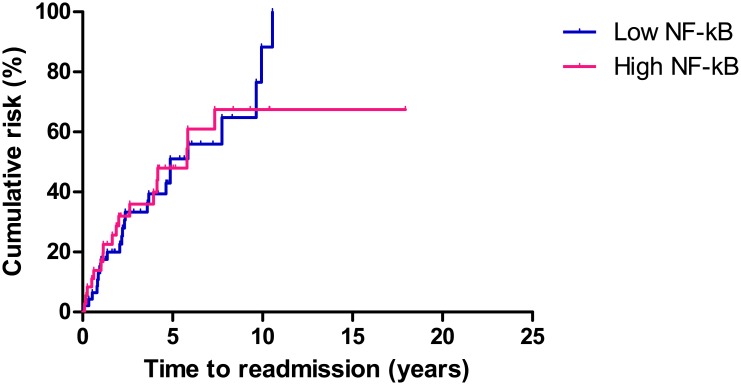
Comparison of readmission in patients with high and low NF-κB activity. There was no significant difference in rates of readmission between the high and low NF-κB activity groups (P = 0.900).

Readmission occurred in 42 (50.6%) patients during the follow-up period. The median time to readmission was 31.3 months (IQR, 15.4–62.7 months) from the primary operation. The cumulative rate of readmission at 1, 5, and 10 years was 13.9%, 41.7%, and 50.0%, respectively, in the patients with high NF-κB activity, and 14.9%, 40.4%, and 48.9%, respectively, in the patients with low NF-κB activity (P = 0.900, Fig 6).

**Fig 7:**
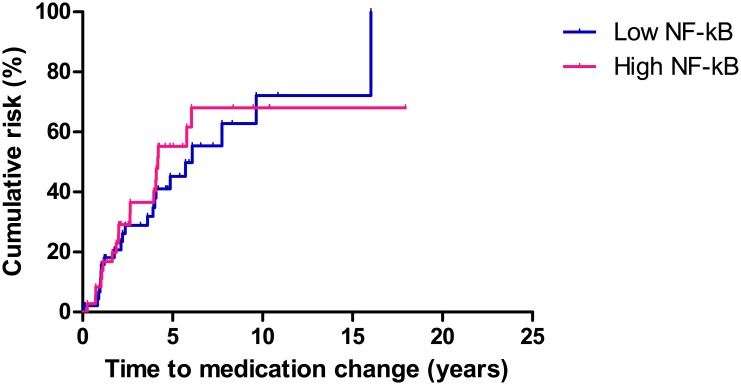
Comparison of medication changes in patients with high and low NF-κB activity. There was no significant difference in rates of medication change between the high and low NF-κB activity groups (P = 0.711).

Medication change occurred in 41(49.4%) patients during the follow-up period. The median time to medication change was 39.1 months (range, 15.7–65.7 months) from the primary operation. The cumulative rate of medication change at 1, 5, and 10 years was 8.3%, 47.2%, and 52.7%, respectively, in the patients with high NF-κB activity, and 10.6%, 36.1%, and 44.7%, respectively, in the patients with low NF-κB activity (P = 0.711, Fig 7).

## Discussion

In the present study, we showed that Crohn’s disease patients with high NF-κB activity had specific clinical manifestations, namely lower frequency of perianal involvement and higher frequency of ileocolonic involvement relative to patients with low NF-κB activity. Total histologic scores were significantly higher in patients with high NF-κB activity than those with low NF-κB activity. However, activation status of NF-κB was not associated with disease outcome after surgery. While many studies have indicated that the NF-κB pathway plays a critical role in inflammatory bowel disease, few studies have correlated NF-κB activation with disease manifestation and prognosis. To the best of our knowledge, this is the first study that evaluated the association between NF-κB activity identified in histologic specimens and clinical characteristics of CD patients who underwent surgical bowel resection.

Our results showed that CD patients with high NF-κB activity display distinctive disease location. While the exact mechanism influencing the difference in disease location and behavior among patients remain to be elucidated, an association between anatomical involvement and clinical features has been reported. A previous study found that perianal disease was more prevalent with colonic involvement than with small intestinal involvement [20], which is consistent with the results in the present work. The additional finding in our study that specific disease location and perianal involvement were associated with NF-κB expression status, may indicate a potential mechanistic explanation relating disease location and behavior among patients.

The possible hypothesis is that high NF-κB activity in bowel reflects high inflammatory burden, thus, results in more extensive ileocolonic involvement. To prove this concept, we compare histologic score with NF-kB activity. Total histologic scores were significantly higher in patients with high NF-κB activity than those with low NF-κB activity, especially polymorphonuclear cell infiltration in lamina propria, nueotrophil infiltration in epithelium, and granuloma were prominent. Furthermore, to verify relationship between NF-κB positivity and systemic inflammation, we reviewed C-reactive protein (CRP) level at the timing of operation. However, this study was retrospective in design, only 37 patients underwent preoperative blood exam for CRP. Due to low statistical power, it was hard to analyze the correlation between NF-κB activity and systemic inflammation. Also, we could not acquire sufficient data for measuring Crohn disease activity index (CDAI), it was impossible to show if NF-κB activity correlates with CDAI.

Risk factors and biomarkers associated with poor clinical outcomes in IBD patients, including smoking, age at diagnosis, history of bowel resection, and circulating obestatin/ghrelin ratio have been reported [21, 22]. In addition, several previous studies also demonstrated that granulomas are associated with poor prognosis [23–26]. As the NF-κB pathway has an important role in the pathogenesis of CD, we hypothesized that overexpression of NF-κB might reflect high inflammatory burden. We therefore evaluated whether CD prognosis was associated with NF-κB expression, but found no significant correlation. A potential reason for this result could be the relatively low number of patients included in the study. With only 83 patients, it is possible that low statistical power limited our ability to detect significant differences between groups. Another possible explanation relates to the fact that we measured NF-κB expression in the resected bowel. After surgical removal of the bowel segment with active disease, the majority of patients went into remission for a period of time. An important prognostic factor for recurrence might be the NF-κB expression in the remnant bowel rather than the resected bowel. According to a previous report, severity of the early postoperative lesion observed on performing ileocolonoscopy had the highest predictive value for postoprerative recurrence [27]. It would therefore also be helpful to measure NF-κB expression in the sample taken from the remnant bowel segment during postoperative surveillance endoscopy.

The present study has several additional limitations. First, we mainly dealt with patients who underwent surgical bowel resection, which raises the possibility of selection bias. Because only 50–71% patients with CD require surgery after diagnosis [28], the patients in our study may not be representative of the full spectrum of CD patients. Second, it is possible that NF-κB expression was heterogeneous in the patients, which could have introduced sampling error. To overcome this problem, we excluded tissue areas accompanying fistulas or perforations and measured NF-κB activation repeatedly in three representative high power fields. Third, while evaluating NF-κB activation by performing immunohistochemistry, we could not measure inflammatory cytokine production, downstream of NF-κB activation. Thus, it was difficult to evaluate the correlation between NF-κB activation, inflammatory cytokine production, and disease prognosis. Previous studies used electrophoretic mobility shift assays (EMSAs) and western blot analysis in cytoplasmic and nuclear colon extracts to measure NF-κB activation. As ours was a retrospective study, we could only use tissues that were already fixed and embedded. The IHC method was ideal for this study because it allowed for measurement of NF-κB activation in relatively old samples. Furthermore, the IHC method using FFPE tissue blocks was easier to perform and more cost-effective, than EMSAs or western blot. Binding of the anti-p65 monoclonal antibody occurs when Inhibitor of κB is released from the NF-κB/I-κB complex. Therefore, the antibody is suitable to identify activated NF-κB *in situ* [29]. We used the microarray method because it was relatively simple and could compare multiple samples at the same time. Therefore, it was both time-effective and cost-effective.

In conclusion, NF-κB activity was associated with specific clinical manifestations in CD patients. Patients with high NF-κB activity showed more frequent ileocolonic involvement and less frequent perianal lesions relative to patients with low NF-κB activity. High NF-κB activity was associated with more severe histologic scores. Further studies are required to establish a causal relationship between NF-κB activity and disease manifestation, and to prove the clinical utility of NF-κB activation status as a disease marker of CD.

## Supporting information

S1 File Dataset. (XLSX)
